# 

**Published:** 1895-11

**Authors:** 


### CONTENTS:

### SELECTIONS.

Article I.—Physical Education. By Harriet M. Lewis, M. D. 289

" II.—What Shall we do with the Temporary Teeth. By Emma Eames Chase, D. D. S. - - - 306

" III.—Regulating. By William J. Brady, D. D. S. - 311

" IV.—Sixth-Year Molars. By N. Pearson, L. D. S. - 314

" V.—Epulis. By A. H. Beers, M. D., D. D. S., L. D. S. 320

" VI.—Habit Spasms. By Dr. E. T. Loeffler. - - 320

### EDITORIAL, ETC.

Why Will Not Fire Burn Brightly When the Sun Shines Upon It? 327

Is Oxygen a Simple Body? - - - - - - - 328

### MONTHLY SUMMARY.

Peroxide of Hydrogen. - - - - - - - - 329

Wedging Teeth with Rubber. - - - - - - - 330

Pulp Protection. - - - - - - - - - 330

Transmission of Cholera by the House Fly. - - - - 331

Varnishing Cavities. - - - - - - - - 332

The First Work of the Dentist. - - - - - - - 332

A Broken Plate-Holder. - - - - - - - - 332

Treatment of Chloroform Asphyxia. - - - - - 333

Cobalt as a Pulp Destroyer. - - - - - - - 333

A Curious Case. - - - - - - - - - 334

Infected Root Canals. - - - - - - - - 334

Cork Wedges for Separating Teeth. - - - - - 334

What Dr. Garret Says of Pyorrhœa Alveolaris. - - - 335

Resetting or Remodeling a Rubber Plate, - - - - 335

Dr. H. A. Wallace. - - - - - - - - - 335

New Anæsthetic Mixture. - - - - - - - 335

Electrolysis in Arresting Erosion. - - - - - 336

To Deodorize Iodoform. - - - - - - - - 336

Smooth Models. - - - - - - - - - 336

Bleaching Teeth. - - - - - - - - - 336

Spirits of Camphor. - - - - - - - - 336

